# Effects of Structured Physical Activity on Motor Fitness in Preschool Children

**DOI:** 10.3390/children11040433

**Published:** 2024-04-05

**Authors:** Filip Kojić, Radenko Arsenijević, Gabrijela Grujić, Lazar Toskić, Jožef Šimenko

**Affiliations:** 1 Faculty of Education, University of Belgrade, 11000 Belgrade, Serbia; filip.kojic@uf.bg.ac.rs (F.K.); gabrijela.grujic@mpn.gov.rs (G.G.); 2 Preschool Teacher Training College Šabac, 15000 Šabac, Serbia; 3 Faculty of Sport and Physical Education, University of Priština in Kosovska Mitrovica, 38220 Leposavić, Serbia; radenko.arsenijevic@pr.ac.rs (R.A.); lazar.toskic@pr.ac.rs (L.T.); 4 Office for Dual Education and National Qualifications for Framework, Ministry of Education, 11000 Belgrade, Serbia; 5 Faculty of Sport, University “Union–Nikola Tesla”, 11000 Belgrade, Serbia; 6 Faculty of Sport, University of Ljubljana, 1000 Ljubljana, Slovenia

**Keywords:** motor coordination, agility, speed of movement, free-play

## Abstract

The aim was to investigate the impact of a specific structured movement activities (SMA) program compared to free play activity (FRP) on the strength, speed, agility, coordination, and balance of motor fitness (MF) in 6-year-old boys and girls. A total of 53 children (24 boys, 29 girls) were randomly allocated to either the SMA group or the FRP group. Both group activities were administered three times a week over a 6-month period. MF variables were assessed before (pre-) and after (post-) using tests: the flamingo balance (FLA), the standing long jump (SLJ), plate tapping (PTT), the obstacle course backwards (OCB), and the shuttle run 4 × 5 m (SRT). At the post-test, the SMA program resulted in significant (*p* < 0.05) improvements in OCB, PTT, SLJ, and SRT tasks. For FRP, a noteworthy improvement was observed only in OCB (ES = 0.45, *p* < 0.05). An ANCOVA revealed a significant group × time interaction (F = 21.71–52.41, η^2^ = 0.258–0.512, *p* < 0.01) for OCB, PTT, and SRT, favoring SMA over FRP. The present findings suggest that SMA may be more effective than FRP when aiming to develop motor coordination, agility, and speed of movement in children.

## 1. Introduction

Developing motor fitness (MF) in children holds significant importance for their overall health and well-being [1]. MF refers to the proficiency and efficiency of motor skills/abilities, including strength, speed, coordination, flexibility, agility, endurance, and balance, and these variables are shown to be closely linked with physical and mental health [2,3,4]. Physical activity serves as a major catalyst in promoting MF, impacting not only children’s physical development but also exerting a profound influence on their cognitive, emotional, and social dimensions [1]. According to the World Health Organization (WHO), it is recommended that children and adolescents aged 5–17 years engage in a minimum of 60 min of moderate-to-vigorous intensity physical activity each [5]. Unfortunately, there is a growing concern among healthcare professionals due to the rising prevalence of sedentary lifestyles and the diminishing physical activity levels observed in children in recent years [5,6]. This trend poses challenges to the holistic development of children, highlighting the need for concerted efforts to encourage and facilitate increased participation in physical activities to promote their MF and overall well-being.

Traditionally, unstructured free-play activities were seen as effective for improving MF in preschool children [7]. However, contemporary research indicates the importance of integrating structured movement activities (SMA) in this age group [7,8]. Particularly, SMA involves teacher-led and organized activities with clear instructions and consistent feedback. In contrast, unstructured activities, such as free play, involve spontaneous whole-body movements, allowing children to expend energy in a freely chosen, enjoyable, and unstructured manner [9,10,11]. In a study conducted by Palmar et al. [12], the engagement of 6-year-old children in physical activity was compared during 30 min of SMA or free play, revealing that children in the SMA engaged in less sedentary behaviors and more in light, moderate and vigorous physical activity. Consistent findings were also reported by other research [13,14] focusing on 5- and 6-year-olds. However, longitudinal studies have presented conflicting findings regarding the effects of SMA and free-play activities on MF in children. In other words, some authors [11,15,16,17,18] observed significant improvement in certain aspects of MF following SMA compared to free play, while others [10,19,20] reported nearly identical changes in motor competence between the two forms of activity. Two possible reasons may account for this discrepancy. Firstly, different SMA prescriptions were utilized across studies (elementary games, ball skill training, mini-sport games, etc.). Secondly, in addition to motor coordination and balance, only a few studies [15,16,21] employed various MF components when exploring the longitudinal effectiveness of SMA. Of note is that their findings revealed pronounced effects on strength and agility in 6-year-old children. In this context, a recent meta-analysis [8] comprising 19 studies concluded that engaging in structured physical exercise has a small but significant impact on the overall MF of preschoolers and that exercise programs with various activities and vigorous intensity may be optimal for children’s motor development.

Despite numerous research efforts, the comparative effectiveness of SMA versus free play in improving specific motor fitness components in preschool children remains unclear. Herein, we implemented a specific SMA program comprising various elementary/relay games, obstacle courses, and dance/music activities. The selection of these contents was informed by prior research indicating that preschool children exhibit the highest engagement level during dance/music activities and elementary/relay games [22,23]. Additionally, obstacle courses were chosen for their potential relevance in enhancing various components of MF [24]. Furthermore, we administered five motor tasks to assess strength, speed, coordination, agility, and balance, aiming to offer a more comprehensive understanding of the effectiveness of SMA on MF in preschool children. Therefore, the current study aimed to investigate the impact of a specific multi-component SMA program compared to free play activity on the diverse aspects of MF in 6-year-old boys and girls.

## 2. Materials and Methods

### 2.1. Experimental Design

**Figure 1:**
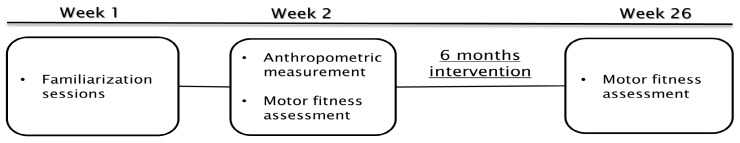
Flowchart of experimental design.

This controlled two-group, repeated-measures experimental study aimed to explore the impact of a specific SMA program on MF in preschool children. Participants were randomly assigned to either the SMA (N = 28) group or the free-play (FRP) (N = 25) group. The randomization process was performed by an online randomizer tool https://www.randomizer.org/ (accessed on 1 April 2023). MF variables were evaluated before and after a 6-month period. All subjects underwent two pre-visits to become acquainted with the motor test, and they were instructed to refrain from physical activity and solid food intake 2 h before testing. The intervention took place during regular school/kindergarten hours to ensure adherence and to assess the feasibility of integrating such a program into the physical education curriculum [25]. Figure 1 illustrates the flowchart of the experimental design.

### 2.2. Subjects

The research was conducted at the beginning of April 2023 on a total sample of 53 preschool children (29 girls, 24 boys, 6.22 ± 0.43 years, height 121.33 ± 4.72 cm, weight 24.41 ± 4.27 kg, body-mass index 16.63 ± 1.59 kg/m^2^). Inclusion and exclusion criteria were as follows: participants needed to be free of musculoskeletal, neurological, and orthopedic disorders and free from diabetes, congenital disorders, or any other metabolic syndrome conditions; the participants were not involved in any additional form of physical exercise outside the kindergarten regular physical education following their curriculum. Informed consent was obtained from the children’s parents/guardians prior to the experiment. Ethical approval (ID 451-03-1/2023-01/4) was obtained from the Faculty of Education, University of Belgrade, and all experiments were conducted in accordance with the Declaration of Helsinki.

#### 2.2.1. Anthropometric Assessment

Morphological variables of body height and body mass were measured. Body height was measured using Martin’s portable anthropometer (Siber-Hegner, Zürich, Switzerland) with an accuracy of 0.1 cm. An electronic scale (Tanita, Arlington Heights, IL, USA) was used to measure the body mass. Afterwards, the body mass index was calculated using the standardized formula proposed by the World Health Organization.

#### 2.2.2. Assessment of Motor Fitness

The assessment of motor skills commenced after a 10 min warm-up session involving light running and warm-up exercises. The assessment protocol, following Eurofit’s standardized procedures [3,26], included five tasks administered in the following order: (i) flamingo balance tests (FLA) for evaluating balance, (ii) plate tapping (PTT) for measuring movement speed, (iii) obstacle course backwards (OCB) for evaluating body coordination, (iv) standing long jump (SLJ) for assessing strength, and (v) shuttle run test 4 × 5 m (SRT) for agility assessment. Each participant had two attempts, and the superior performance was recorded for further analysis. Two experienced physical education teachers supervised the entire process to ensure proper form.

In the FLA test, participants stood on one fully stretched leg on a specialized wooden beam (50 × 3 × 4 cm), flexed the free leg at the knee, and held the foot with the hand on the same side. Their task was to maintain the position for as long as possible, with the test concluding if they touched the other leg, the floor, or sustained balance for over 60 s. Results were based on the duration (in seconds) spent in balance on one leg.

The SLJ test required participants to stand with feet shoulder-width apart and jump forward as far as possible, landing on both feet. The distance (in centimeters) from the starting line to the back of the heel closest to the starting line determined the score.

For the PTT, participants sat at a wooden table with two circles measuring 20 cm in diameter. They placed their non-dominant hand in the middle of the table and their dominant hand on one circle, crossing over the weaker hand. The goal was to rapidly tap the circles alternately with the fingers of the dominant hand for 20 s.

The OCB involved moving on all fours (supported only on feet and palms) backwards over a 10 m distance. Two obstacles were set, requiring climbing over the vaulting box cover and crawling under the vaulting box frame (3 and 6 m from the starting line, respectively).

In the SRT, participants sprinted and made turns at maximum speed between two parallel lines spaced 5 m apart. Two assistants were stationed at both ends, and participants touched their hands (positioned behind the line) before swiftly returning at maximum speed.

#### 2.2.3. Experimental Intervention

Both SMA and FRP activities were scheduled three times per week over a six-month period. We opted for three sessions per week instead of two, based on previous findings suggesting that engaging in organized physical activity twice weekly might not be sufficient to stimulate motor function improvement in preschoolers [8]. Both activities (i.e., SMA and free-play) took place outdoors and in the institutional gymnasium, depending on the prevailing weather conditions. Each session lasted 35 min and took place in the morning hours (8–11 a.m.). The SMA program encompassed a diverse range of activities, including elementary/relay games (competitive, tag, and team games), obstacle courses (consisting of 9–12 obstacles with a wide variety of movements like running, jumping, turning, crawling, passing, etc.), and dance/music activities (involving both ethnic and modern dance choreographies, as well as musical games). Prior to each type of activity, a specific warm-up was conducted, following methodological guidelines for preschoolers’ physical education [27]. Specific activities were designated for each day (Monday/Wednesday/Friday), and these were organized through frontal, group, and circuit formats. The intensity of activities was adjusted based on observable external signs such as sweat, blush, and spontaneous breaks. Additionally, heart rates were considered, using a pulse meter attached to five randomly selected children at the beginning of the study, with the maximum falling between 170 and 180 beats per minute [15]. Note that heart rate monitoring was used only to monitor the intensity of the experiment. An example of a weekly SMA routine is detailed in Table 1.

### 2.3. Statistical Analysis

Prior to analysis, data were checked for normality using the Kolmogorov–Smirnov Test. All tests for both groups have shown normal distribution.

One-way ANCOVA (using baseline values as covariates) was used to examine differences in the tested variables, between the SMA and FRP groups in the post-test values. If ANCOVA showed statistical significance, the Bonferroni post-hoc test was used for further estimation of differences between groups [28]. Differences in the baseline values between groups were analyzed by the independent samples *t*-test. Differences in the pre- to post-values, of each test (SLJ, FLA, PTT, AGI, and OCB), and for each experimental group independently (SMA and FRP), were determined by the paired samples *t*-test.

The eta squared (η^2^) was calculated for the ANCOVAs with the following classification for magnitude effects [29]: no effect (η^2^ < 0.04), minimum effect (0.04 < η^2^ < 0.25), moderate effect (0.25 < η^2^ < 0.64), and strong effect (η^2^ > 0.64). However, the magnitude of difference [30] was tested by means of effect size (ES), in a case of using paired samples *t*-test, where the difference was considered either very small (0.01), small (0.2), moderate (0.5), large (0.8), very large (1.2) or huge (larger than 2.0) for the post-hoc test [31]. A significant level of *p* < 0.05 was used for all comparisons [32]. All statistical procedures were performed by SPSS version 20.0 (SPSS Inc., Chicago, IL, USA) and Microsoft Office Excel 2016 (Microsoft Corporation, Redmond, WA, USA). All data are available in Supplementary Materials. 

## 3. Results

The implemented experiment’s descriptive statistics and ANCOVA results are provided in Table 2.

**Figure 2:**
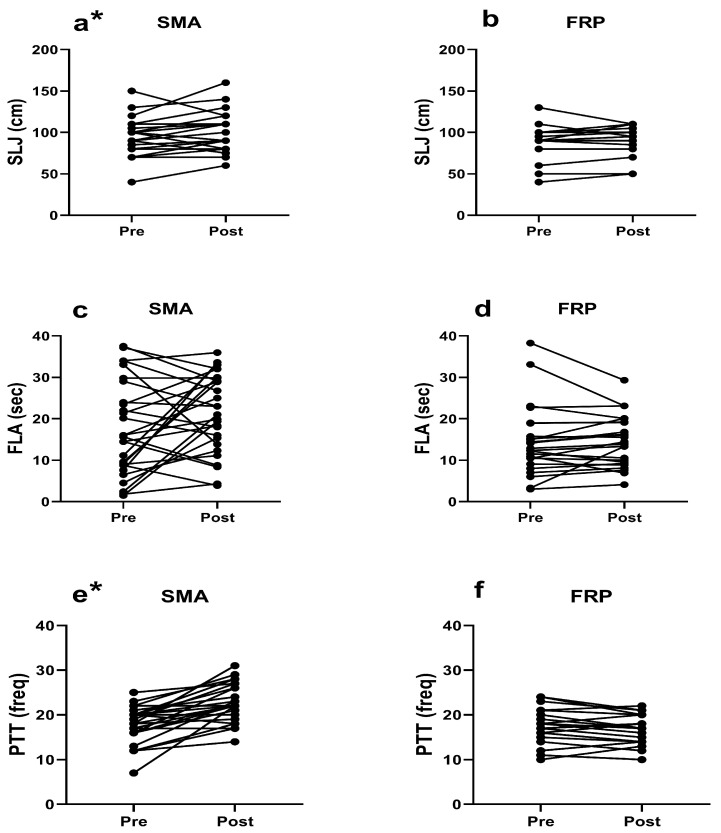
Individual trend of changes before (pre) and after (post) of 6 months of different school activities, for the following variables: standing long jump test (SLJ) (**a**) for structured movement activities group (SMA), (**b**) and free-play group (FRP); flamingo test (FLA) (**c**) for SMA, (**d**) for FRP; plate tapping test (PTT) (**e**) for SMA, (**f**) for FRP; * significantly different pre- to post-test using paired samples *t*-test (*p* < 0.05).

*Standing long jump test (SLJ)*. Individual trends of changes are shown in Figure 2a for the SMA group, and in Figure 2b for the FRP group. No significant baseline differences between the SMA and FRP groups were observed (*p* = 0.411). The SMA group demonstrated a significant improvement in SLJ relative to the pre-test by 7.21% (6.79 ± 1.35 cm, *p* = 0.024, ES = 0.45), whereas the FRP group showed no significant differences between post- and pre-test, but an increase of 1.34% (1.20 ± 2.20 cm, *p* = 0.442, ES = 0.16) was observed. Furthermore, ANCOVA did not reach significance in the post-test comparison between SMA and FRP, though significance was very close to being achieved (*p* = 0.052).

*Flamingo test (FLA)*. Individual trends of changes are presented in Figure 2c for the SMA group and Figure 2d for the FRP group. Similarly, no significant baseline differences were found between the two groups (*p* = 0.238). Neither group demonstrated significant changes relative to the pre-test (SMA by 21.73%: 3.84 ± 1.96 s, *p* = 0.078, ES = 0.35; FRP by 1.10%: 0.16 ± 2.21 s, *p* = 0.844, ES = 0.20). However, ANCOVA showed significance in the post-test in favor of the SMA (by 6.89 ± 3.33 s, *p* = 0.005), showing minimum effect size magnitude (η^2^ = 0.148).

*Plate tapping test (PTT)*. Descriptive data of individual trends of changes is shown in Figure 2e for the SMA group, and in Figure 2f for the FRP group. No significant differences in the baseline measurement between SMA and FRP were found (*p* = 0.279). The SMA group showed significantly increased post-test values relative to the pre-test, by 29.42% (5.29 ± 0.47 freq, *p* = 0.000, ES = 1.24), whereas this was not accomplished in the FRP group, with a decrease of 2.86% (0.48 ± 0.67 freq, *p* = 0.238, ES = 0.24). ANCOVA achieved significant differences in the post-test, with SMA showing higher PTT values by 29.98% and a moderate effect (6.97 ± 1.05 freq, *p* = 0.000, η^2^ = 0.512).

**Figure 3:**
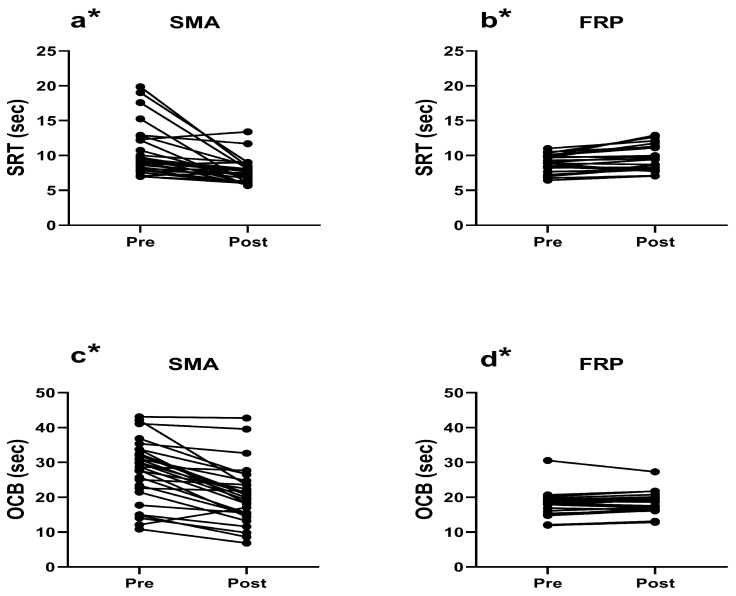
Individual trend of changes before (pre) and after (post) of 6 months of different school activities, for the following variables: agility test (SRT) (**a**) for structured movement activities group (SMA), (**b**) and free-play group (FRP); obstacle course backwards test (OCB) (**c**) for SMA, (**d**) for FRP; * significantly different pre- to post-test using paired samples *t*-test (*p* < 0.05).

*Shuttle ran agility test (SRT).* Individual trends of changes are presented in Figure 3a for the SMA group and Figure 3b for the FRP group. In contrast to all previous variables, significant baseline differences were found between SMA and FRP (*p* = 0.025). SMA values significantly decreased from pre- to post-test by 27.15% (2.86 ± 1.82 s, *p* = 0.000, ES = 0.84). Conversely, for FRP, pre-test achieved significantly lower values than post-test by 7.66% (0.68 ± 0.49 s, *p* = 0.002, ES = 0.71). ANCOVA showed significance in the post-test, with lower values for SMA relative to FRP by 24.19% (1.86 ± 0.05 s, *p* = 0.000), and a moderate effect size (η^2^ = 0.328).

*Obstacle course backwards test (OCB)*. Individual trends of changes are displayed in Figure 3c for the SMA group and Figure 3d for the FRP group. Significant baseline differences were established between SMA and FRP (*p* = 0.000). Paired samples *t*-test for SMA showed significance, with post-test values lower than pre-test by 24.75% (6.79 ± 0.56 s, *p* = 0.000, ES = 1.29). Concerning FRP, pre-test values were significantly lower than post-test values by 2.88% (0.51 ± 0.56 s, *p* = 0.028, ES = 0.47). Nevertheless, ANCOVA succeeded in reaching significance in post-test, with SMA lower values than FRP by 11.98% (2.47 ± 5.08 s, *p* = 0.000), and a moderate effect size magnitude (η^2^ = 0.258).

## 4. Discussion

MF serves as an important health indicator in children. This study sought to compare the impacts of six months of SMA and unstructured free-play activities on various MF components (coordination, strength, speed, agility, and balance) in six-year-old children. The primary findings demonstrated a significant increase in motor coordination, agility, and speed of movement after SMA, with the enhancement being notably greater compared to the effects of free-play activities (for details, see Table 2). In contrast, free-play activities showed a positive effect, specifically on motor coordination.

Current results suggest that SMA intervention led to notable improvements in three of the five evaluated components of MF. Specifically, motor coordination, agility, and speed of movement exhibited significant changes post-SMA intervention, while the increase in muscular strength approached statistical significance. Notably, only alterations in balance were out of significance, although the SMA group demonstrated superior performance in the post-FLA test compared to the FRP group. Nevertheless, current results suggest that the SMA program was highly effective in improving various MF components among preschoolers. A recent meta-analysis [8] concluded that engaging in structured physical exercise has a small but significant impact on the overall MF of preschoolers, with a particular emphasis on improvements in muscular strength and agility. Despite not reaching statistical significance for SLJ, the small effect size (ES = 0.45) observed in the current study aligns with previous reports [15,16,21]. Moreover, the current findings support previous notions [15,16] that SMA intervention elicits a moderate-to-large effect on agility gains. The relatively greater ES observed for agility compared to strength gains (ES = 0.84 vs. 0.48) in the current study may be attributed to the specific SMA content. There is a general assumption that specific training affects the development of some MF components while having no effect on others. In other words, engaging in specific task-oriented practice is believed to directly impact that particular task, without necessarily affecting other tasks related to the same competency [20,33]. In the present study, various types of elementary/relay games were incorporated into the SMA program, relying largely on fast change of direction maneuvers. Given that performance in the agility-SRT test highly depends on directional changes in movement [34], the inclusion of tag, competitive, and team games in the SMA program is partially expected to be beneficial for improved SRT test results. On the other hand, it could be speculated that a more strength-oriented SMA program (i.e., incorporating strength exercises) may be advantageous for greater strength gains [15,21].

The current experimental program demonstrated the most substantial post-test effects (ES = 1.24–1.29) on OCB and PTT variables, indicating a superior enhancement in motor coordination and speed of movement. The observed enhancement in speed of movement aligns well with the findings of [18], where a 4-month SMA program involving tag and relay games, as well as sports games and skills, led to noteworthy improvements in PTT performance among 6-year-old boys and girls. On the other hand, the present findings are particularly relevant in light of conflicting reports on the effectiveness of SMA programs in improving coordination in children. For instance, Abusleme-Allimant and co-workers [10] utilized the Test of Gross Motor Development (TGMD) to assess motor competence in 6-year-old children and found similar improvements in TGMD performance following 12 weeks of SMA (one session per week including imitation games, circuit training, and ball-skill training) and free-play activities. Comparable results were reported by [20] where a 10-week SMA program (one session per week including manual dexterity, balance, and mobility exercises) did not elicit beneficial improvement on TGMD in 4–5-year-old children compared to free-play activities. Several factors may contribute to the inconsistencies in findings between the current study and previous research on motor coordination. Specifically, the variance in total time of intervention (6 months vs. ≤3 months), weekly frequency of practice (3 sessions/week vs. 1–2 sessions/week) and motor test utilized to access coordination (OCB test vs. TGMD) across studies could impact outcome results. In fact, the current literature suggests a minimum intervention duration of at least 6 months [21,35] with three sessions per week [8] for successful kindergarten-based interventions, and some of the previous reports do not align with these recommendations. Another significant factor contributing to discrepancies between studies may be related to the specific SMA prescription. Herein, we incorporated multiactivity content into the SMA program, which showed to be well-suited for coordination development. This may be particularly related to the obstacle course sessions (conducted once weekly), considering recent findings [24] that obstacle course-based intervention programs may be highly effective in enhancing motor competence among 6- to 7-year-old Flemish children. Furthermore, our SMA program included dance and musical activities involving intricate choreography, footwork, and arm movements, requiring children to synchronize their actions with the music. This synchronization enhances their spatial awareness [36] and may promote the development of MF. Additionally, the repetitive nature of dance sequences aids in reinforcing neural connections, thereby improving motor coordination [37].

Finally, it is important to acknowledge some limitations of this study. Firstly, there was a lack of monitoring of the children’s morphological status during the experiment apart from body mass and height. Additionally, participants’ dietary habits were also not monitored, constituting a second limitation. The next limitation is the heart rate monitoring. Heart rate monitoring devices were not available for every participant in the experiment, and they were rotated on a weekly basis between participants. Baseline differences have shown undesirable significance in the two variables SRT and OCB, which is one of the limitations of the random sampling method that might occur. Lastly, the study did not track the children’s daily unstructured activities and periods of inactivity, potentially influencing fitness levels and the development of abilities, serving as a third limitation. The future studies might take these factors into account. Furthermore, the interpretation and main findings of the present study’s results may not be representative of children with varying backgrounds, abilities, or health conditions, as all participants were free of musculoskeletal, neurological, orthopedic disorders, diabetes, congenital disorders, or any other metabolic syndrome conditions.

## 5. Conclusions

In conclusion, the present study highlights the importance of choosing adequate activity in which 6-year-old children were engaged. Additionally, the obtained findings explicitly suggest that SMA might be more appropriate in comparison with FRP when we aim to develop motor coordination, agility, and speed of movement in the above-mentioned age group. Specifically, applied SMA involves organized activities that are constantly monitored by teachers, and teachers need to provide clear instructions and external feedback to preschool children. In contrast, FRP contains natural whole-body movements, which let preschool children let out energy in a free style. Therefore, organized teacher-monitored activities such as SMA could provide the aforementioned benefits. Finally, it could be concluded that SMA undoubtedly demonstrated potential for further application in a population of preschool children, and that it is necessary to further explore this type of activity in other populations of children, and to compare it with other forms of the activity.

## Figures and Tables

**Table 1:** Example of a weekly SMA routine.

Monday	Wednesday	Friday
Tag game (5 min) Warm-up exercises (5 min)	Various movements with changeable speed (5 min) Warm-up exercises (5 min)	Musical game (5 min)
Relay race (20 min)	Obstacle course (20 min)	Dance choreography (25 min)
Cool-down (5 min)	Cool-down (5 min)	Cool-down (5 min)

**Table 2:** Results of descriptive statistics (mean ± sd) of pre- to post-test, ANCOVA, and baseline measurements of the MF variables.

	SMA		FRP		*p* (ANCOVA)	F (ANCOVA)
	Pre	Post	Pre	Post		
SLJ	94.11 ± 21.35	100.58 ± 22.69	89.40 ± 20.02	90.60 ± 17.81	0.052	3.966
FLA	17.65 ± 11.29	21.49 ± 9.34	14.44 ± 8.21	14.60 ± 6.01	0.005 *	8.717
PTT	17.96 ± 3.95	23.25 ± 4.42	16.76 ± 4.04	16.28 ± 3.37	0.000 *	52.414
SRT	10.54 ± 3.55 ^#^	3.55± 1.73	8.86 ± 1.30	9.54 ± 1.79	0.000 *	24.417
OCB	28.04 ± 10.36 ^#^	21.18 ± 9.96	17.66 ± 3.83	18.17 ± 3.27	0.000 *	21.709

MF—motor fitness; SMA—structured movement activities; FRP—free-play; SLJ—standing long jump test; FLA—flamingo test; PTT—plate tapping test; SRT—shuttle run agility test; OCB—obstacle course backwards test; *—significant difference in pre-post values; ^#^—significant difference in baseline measurements between groups.

## Data Availability

Data are contained within the article and Supplementary Materials.

## Supplementary Materials

The following supporting information can be downloaded at: https://www.mdpi.com/article/10.3390/children11040433/s1, File S1: Study dataset.
